# ZIF-67-Derived CoSe/NC Composites as Anode Materials for Lithium-Ion Batteries

**DOI:** 10.1186/s11671-019-3194-5

**Published:** 2019-12-02

**Authors:** Zongyang Li, Lian Ying Zhang, Lei Zhang, Jiamu Huang, Hongdong Liu

**Affiliations:** 1 Chongqing Key Laboratory of Micro/Nano Materials Engineering and Technology, Chongqing University of Arts and Sciences, Chongqing, 402160 People’s Republic of China; 2 Research Institute for New Materials Technology, Chongqing University of Arts and Sciences, Chongqing, 402160 People’s Republic of China; 3 College of Materials Science and Engineering, Chongqing University, Chongqing, 400045 People’s Republic of China; 4 Institute of Materials for Energy and Environment, Qingdao University, Shandong, 266071 People’s Republic of China; 5 College of Life Science, Chongqing Normal University, Chongqing, 401331 People’s Republic of China

**Keywords:** Lithium-ion batteries, Anode materials, CoSe, ZIF-67

## Abstract

As a typical metal selenide, CoSe is a kind of foreground anode material for lithium-ion batteries (LIBs) because of its two-dimensional layer structure, good electrical conductivity, and high theoretical capacity. In this work, the original CoSe/N-doped carbon (CoSe/NC) composites were synthesized using ZIF-67 as a precursor, in which the CoSe nanoparticles are encapsulated in NC nanolayers and they are connected through C–Se bonds. The coating structure and strong chemical coupling make the NC nanolayers could better effectively enhance the lithium storage properties of CoSe/NC composites. As a consequence, the CoSe/NC composites deliver a reversible capacity of 310.11 mAh g^−1^ after 500 cycles at 1.0 A g^−1^. Besides, the CoSe/NC composites show a distinct incremental behavior of capacity.

## Background

With the depletion of fossil energy represented by petroleum and the increasing environmental pollution caused by the burning of fossil fuels, there is an urgent need for a sustainable renewable energy source. Lithium-ion batteries (LIBs) stand out from many new energy sources because of their high energy density, long cycle life, and environmental friendliness [1, 2]. They are widely used in mobile electronic devices and electric vehicles. However, the anode material of commercial LIBs is graphite, and the theoretical capacity of graphite is only 372 mAh g^−1^, which cannot meet the capacity requirements of large-scale electronic equipment such as electric vehicles, and limits the application and development of LIBs [3–6]. Therefore, scientists have developed a variety of anode materials to increase the capacity and rate performance of LIBs, such as carbon materials [7–9], transition metal oxides [10–13], metal sulfides [14–17], metal phosphides [18–21], and metal selenide [22–25].

Selenium has higher density and conductivity, so metal selenide has higher energy density and rate performance than transition metal oxides and sulfides [26]. Compared with widely studied oxides and sulfides, selenide has been relatively rare in the field of LIBs. Among selenides, CoSe is regarded as an excellent anode material for LIBs because of its two-dimensional layer structure, good electrical conductivity, and high theoretical capacity [22]. However, as a negative electrode material for lithium storage based on conversion reaction, like transition metal oxides and sulfides, CoSe suffers serious volume expansion during charge and discharge, resulting in the breakage and pulverization of active materials and loss the connection with the current collector, further causing the acute capacity attenuation [10]. According to previous literature [25, 26], preparing nanostructures and recombining with carbon materials can effectively relieve the abovementioned problems. The porous nanostructures are advantageous to the permeation of electrolyte within electrode materials and shorten the diffusion of lithium ions. Meanwhile, the porous structure could supply free space for volume expansion to prevent the destruction of structure, which enhances the cyclic stability. Besides, recombining metal compounds with carbon materials could fully utilize the excellent conductivity and mechanical property of carbon materials to improve the conductivity of metal compounds and buffer the strain caused by volume expansion, which is favorable to the rate and cycling performance. However, at present, metal compounds and carbonaceous matrix are connected through physical adsorption. Compared with strong coupling, such as chemical bonds, the weak connection will make the metal compounds nanoparticles fall off from carbonaceous matrix under the condition of large current density and long-term cycle [4, 22]. The construction of strong coupling between metal compounds and carbonaceous matrix is still a challenge.

Metal-organic frameworks (MOFs) are a class of porous materials formed by the attachment of metal ions to organic compounds via coordinate bonds [27–30]. Because of its porous structure, high specific surface area, and structural controllability, it has broad application prospects in gas storage separation, catalysis, sensors, and drug transportation [31, 32]. ZIF-67 is a typical Co-based MOFs material formed by Co^2+^ and 2-methylimidazole and has a porous structure similar to zeolite. 2-Methylimidazole has a nitrogen-containing functional group that carbonizes to form N-doped carbon (NC) through pyrolysis [9, 33]. On the one hand, NC can alleviate volume expansion and improve cycle stability; on the other hand, doping of N atoms can improve conductivity while reflecting the active site and increasing its lithium storage capacity [8]. Besides, the materials derived from MOFs could remain the porous structure. Therefore, ZIF-67 is often used as a precursor to prepare composite materials of cobalt-based compounds such as Co_3_O_4_ [34, 35], CoS [36, 37], CoP [38–40], and N-doped carbon.

**Fig. 1:**
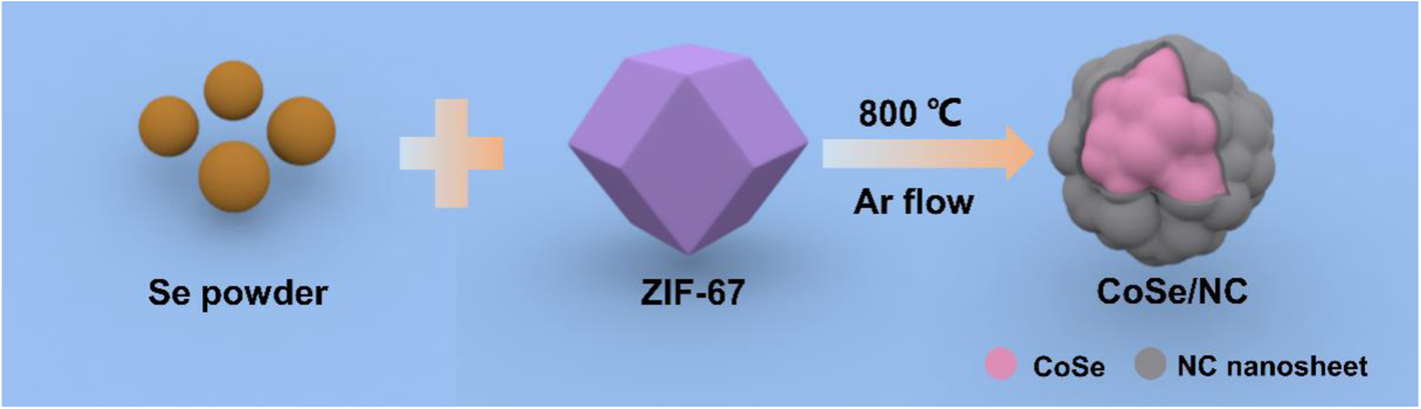
Schematic diagram for the synthesis of CoSe/NC composites

Here, we report a facile method to prepare CoSe/N-doped carbon (CoSe/NC) composites through the selenization of ZIF-67 with selenium powder at inert atmosphere. Figure 1 shows the synthesis process of CoSe/NC composites. In the selenization process, the Co^2+^ within ZIF-67 reacts with selenium to form CoSe nanoparticles, which are enfolded by NC nanolayers that originate from the carbonization of 2-methylimidazole. The NC nanolayers could enhance the conductivity of CoSe and suppress the volume expansion, and offer additional lithium storage as active sits. The porous structure from ZIF-67 could shorten the diffusion path of electrons and lithium ions. More importantly, the C–Se bonds between CoSe nanoparticles and NC nanolayers; the unique chemical connection could not only better promote the electrical active connection between CoSe and NC, but also better mitigate the volume variation. As a result, the CoSe/NC composites show superb lithium storage properties.

## Methods

### Preparation of ZIF-67

All chemicals are analytical grade and used without further purification. In a typical synthesis, 1.436 g of Co(NO_3_)_2_·6H_2_O and 3.244 g of 2-methylimidazole were dissolved in 100 ml of methanol solution, respectively. Subsequently, the Co(NO_3_)_2_·6H_2_O solution was poured into 2-methylimidazole solution with stirring for 12 min, and then aged for 20 h. Finally, the resulting purple precipitates were collected by centrifugation, washed with methanol several times, and dried in air at 60 °C.

### Preparation of CoSe/NC

The CoSe/NC was prepared by the selenization of ZIF-67 with selenium powder. In a typical synthesis, ZIF-67 and selenium powder were mixed with a mass ratio of 1:1. Subsequently, the mixing powder was placed in a ceramic boat at the tube furnace under Ar atmosphere and heated to 800 °C for 4 h with a heating speed of 2 °C min^−1^. To compare, pure CoSe was prepared through similar procedure except using cobalt powder as starting materials.

### Material Characterization

The powder XRD patterns of the samples were obtained on a TD-3500X X-ray diffractometer with Cu Kα radiation (*λ* = 1.5418 Å) at a scan rate of 0.05 s^−1^. The Raman spectrums were recorded on a LabRAM HR800 Raman spectrometer with 532-nm laser light. TGA was carried out on a STA 449 F3 thermoanalyser under ambient atmosphere and at a heating rate of 10 °C min^−1^ from ambient temperature to 900 °C. XPS measurements were performed on a Thermo ESCALAB 250XI X-ray photoelectron spectrometer with monochromatic Al Kα radiation (hν = 1486.6 eV). Nitrogen adsorption/desorption isotherms were collected at 77 K using a BELSORP-Max instrument, which were used to evaluate the specific surface area (BET) and pore size distribution (BJH). FESEM images were collected through Quanta 250 scanning electron microscopy and SIGMA 500 scanning electron microscopy, respectively. TEM images and SAED were taken on a Tacnai G2 F20 transmission electron microscopy.

### Electrochemical Measurements

All electrochemical behaviors of as-synthesized samples were performed using CR2032 coin-type cell. The working electrodes were prepared by mixing 80 wt% of active materials, 10 wt% of acetylene black, and 10 wt% of polyvinylidene fluoride (PVDF) binder in an appropriate *N*-methyl-2-pyrrolidone (NMP) to form a slurry. Subsequently, the slurry was uniformly coated on Cu foil and dried at 80 °C for 4 h in air and then punched in disks with a dimeter of 14 mm and dried at 120 °C for 12 h under vacuum. The metallic lithium foil was used as both counter electrode and reference electrode. The electrolyte was 1.00 M LiPF_6_ in ethylene carbonate and diethyl carbonate (EC: DEC = 1:1) and the separator was Celgard 2500 film. The all cells were assembled in an argon-filled glovebox with the content of oxygen and moisture below 1 ppm. The galvanostatic charge-discharge tests were conducted on a Neware CT-3008W battery test system between 0.01 and 3.0 V at room temperature. The cyclic voltammetry (CV) measurements between 0.01 and 3.0 V at a scan rate of 0.2 mV s^−1^ and electrochemical impedance spectroscopy (EIS) with the frequency range from 0.01 to 100 kHz were all performed on a CHI 760E electrochemical workstation.

## Results and Discussion

**Fig. 2:**
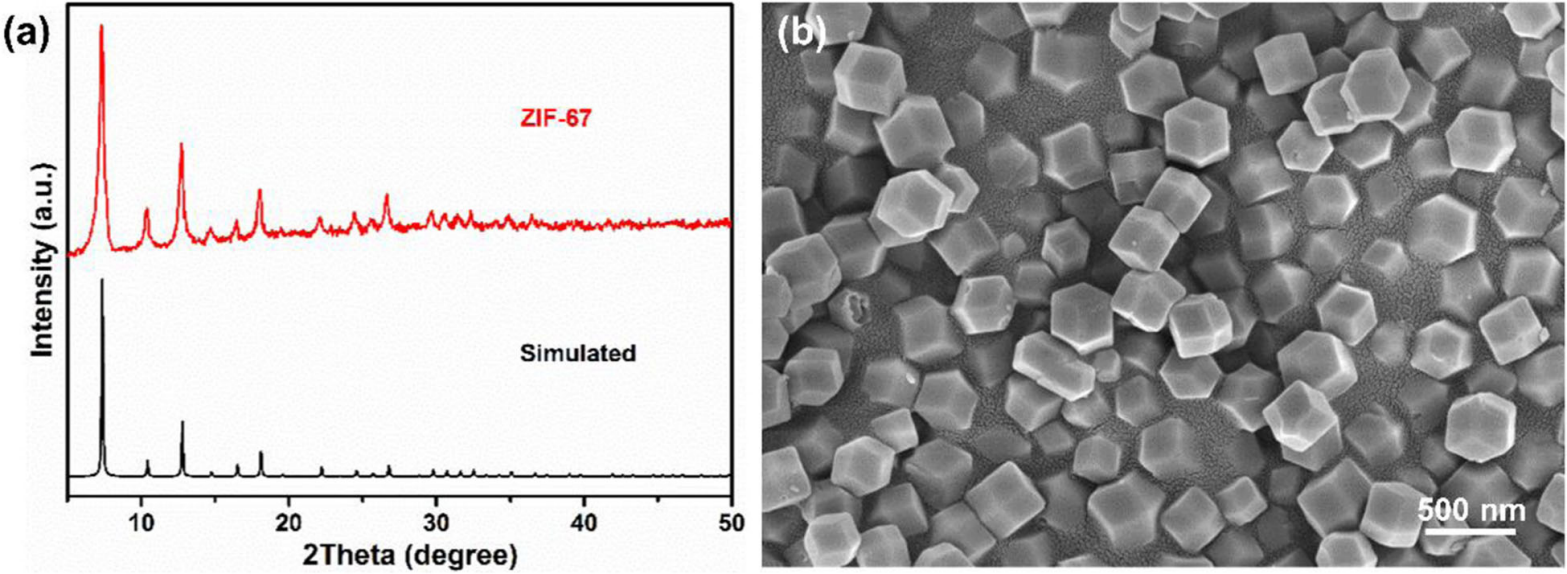
**a** XRD patterns of as-synthesized ZIF-67 and simulated XRD patterns of ZIF-67. **b** SEM image of as-synthesized ZIF-67

The crystal structure and morphology of as-synthesized ZIF-67 were confirmed by XRD and SEM, respectively, as shown in Fig. 2. The diffraction pattern of ZIF-67 is identical with simulated pattern and previous reports (Fig. 2a) [41, 42]. The ZIF-67 exhibits a decahedral morphology with a size of 300 nm, which could be seen from the SEM image (Fig. 2b and Additional file 1: Figure S1). Meanwhile, the ZIF-67 displays a purple appearance that similar with previous synthesis. These results show the successful synthesis of ZIF-67.

**Fig. 3:**
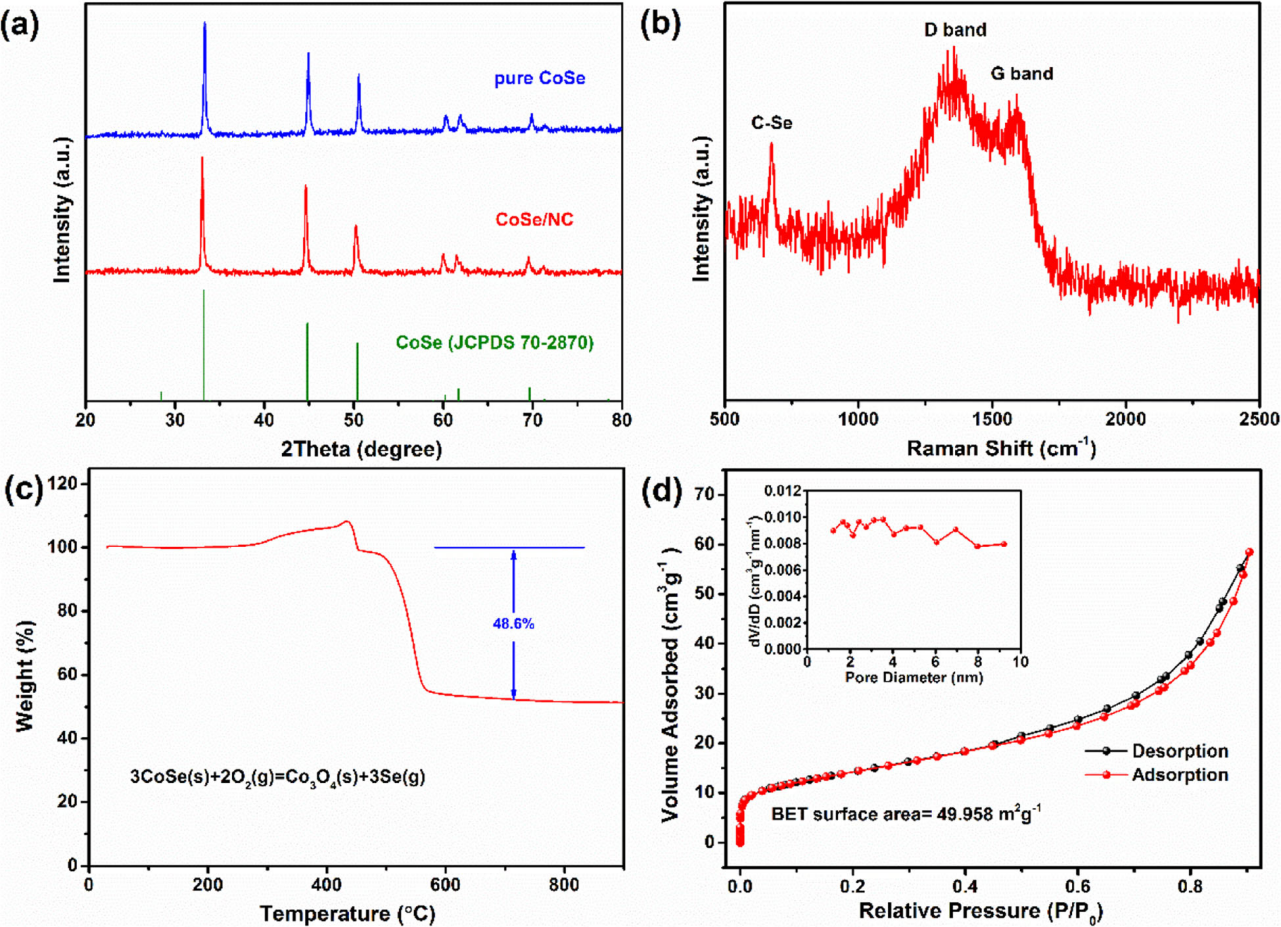
**a** XRD patterns of CoSe/NC and pure CoSe. **b** Raman spectrums of CoSe/NC. **c** TGA curve of CoSe/NC. **d** Nitrogen adsorption-desorption isotherm and diameter distribution profiles of CoSe/NC.

Figure 3a presents the XRD patterns of CoSe/NC and pure CoSe, in which all peaks can be assigned to CoSe (JCPDS 70-2870). Besides, the peaks are intense and no other peaks can be observed, suggesting the high crystalline and purity. However, there is no hump or peak of carbon materials in XRD pattern of CoSe/NC, which could be related with graphitization degree and content of carbon. To confirm the presence of carbon in CoSe/NC, the Raman spectra were obtained. As can be seen from Fig. 3b, two broad peaks at 1360 and 1590 cm^−1^ are severally attributed to the defects (D-band) and ordered graphitic carbon (G-band), indicating the presence of carbon materials in CoSe/NC [8]. The value of ID/IG is 1.05, suggesting the carbon materials with rich defects. Besides, there is a sharp and strong peak at 675 cm^−1^, which is related with C–Se bond [22, 43]. The presence of C–Se bond indicates the connection between CoSe and NC is not common physical adsorption; instead, it is a kind of chemical connection through C–Se bond. Compared with physical adsorption, the unique chemical connection could not only better promote the electrical active connection between CoSe and NC, but also better mitigate the volume variation. The TGA was used to determine the NC content in CoSe/NC. According to the TGA curve and chemical equation (Fig. 3c), the mass percent of NC in CoSe/NC is evaluated to be 11.7%. Figure 3d shows the N_2_ adsorption/desorption isotherm of CoSe/NC with a typical type-IV isotherm and a type-H3 hysteresis loop, suggesting the mesoporous feature. The CoSe/NC exhibits a large specific surface area (BET) of 49.958 m^2^ g^−1^. However, the specific surface area of CoSe/NC is far less than that of the precursor ZIF-67, which may be caused by the structure collapse of ZIF-67 during calcination process and the volume expansion due to the formation of CoSe. The pore size distribution (BJH) displays the primary pores in a range of 1 to 10 nm with an average size of 7.238 nm. The large specific surface area and mesoporous structure are advantageous to the penetration of electrolyte and the fast transport of Li^+^.

**Fig. 4:**
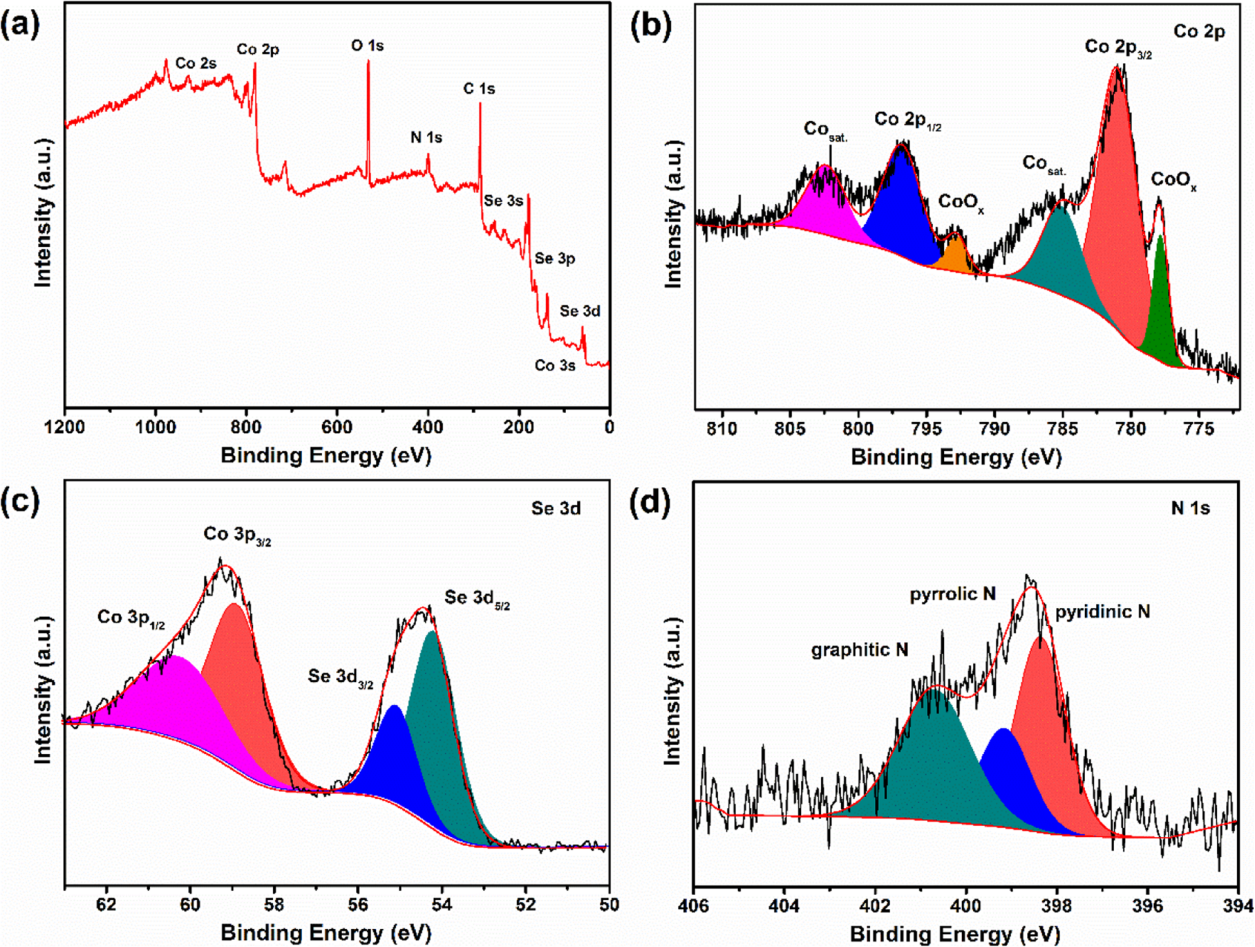
XPS spectrums of CoSe/NC: **a** survey, **b** Co 2p, **c** Se 3d, and **d** N 1s

XPS was introduced to characterize the elemental component and valence state in CoSe/NC, as shown in Fig. 4. The survey of CoSe/NC shows the presence of Co, Se, C, N, and O elements (Fig. 4a). The characteristic peaks of Co 2p_1/2_ and Co 2p_3/2_ could be observed at 796.92 and 780.94 eV in Co 2p spectrum, which could be assigned to CoSe (Fig. 4b). The two peaks located at 785.55 and 802.53 eV are the shakeup satellites of Co^2+^ [22, 44, 45]. Besides, the other two peaks at 793.03 and 778 eV are probably related with CoO_x_, which is caused by the oxidized environment. The Se 3d spectrum displays two peaks at 54.94 and 54.08 eV, which could be ascribed to Se 3d_3/2_ and Se 3d_5/2_, respectively (Fig. 4c). The C 1s spectrum (Additional file 1: Figure S2) exhibits three peaks, which could be indexed to C 1s, Nsp^2^-C, and Nsp^3^-C, respectively. The N 1s spectrum in Fig. 4d exhibits graphitic N peak at 287.6 eV, pyrrole N peak at 286 eV, and pyridine N peak at 284.7 eV, respectively, indicating that the organic linker was converted to nitrogen-doped carbon through calcination [8, 46]. The results are identical with C 1s spectrum. Besides, the quantitative analysis of XPS was conducted. The results are shown in Table 1, which suggests that the nitrogen content in CoSe/NC is 12.08% (atomic %) and the contents of graphitic N, pyrrole N, and pyridine N are 35.02%, 37.46%, and 27.52% (atomic %), respectively. Based on the previous reports, on the one hand, the doped nitrogen is beneficial to enhance the conductivity; on the other hand, the pyrrole N and pyridine N can increase the storage of lithium as electrochemically active site.

**Table 1:** Percentage of nitrogen in CoSe/NC and different types of N in total nitrogen

Nitrogen in CoSe/NC (atomic %)	In total N (atomic %)		
	Graphitic N	Pyrrolic N	Pyridinic N
12.08	35.02	37.46	27.52

**Fig. 5:**
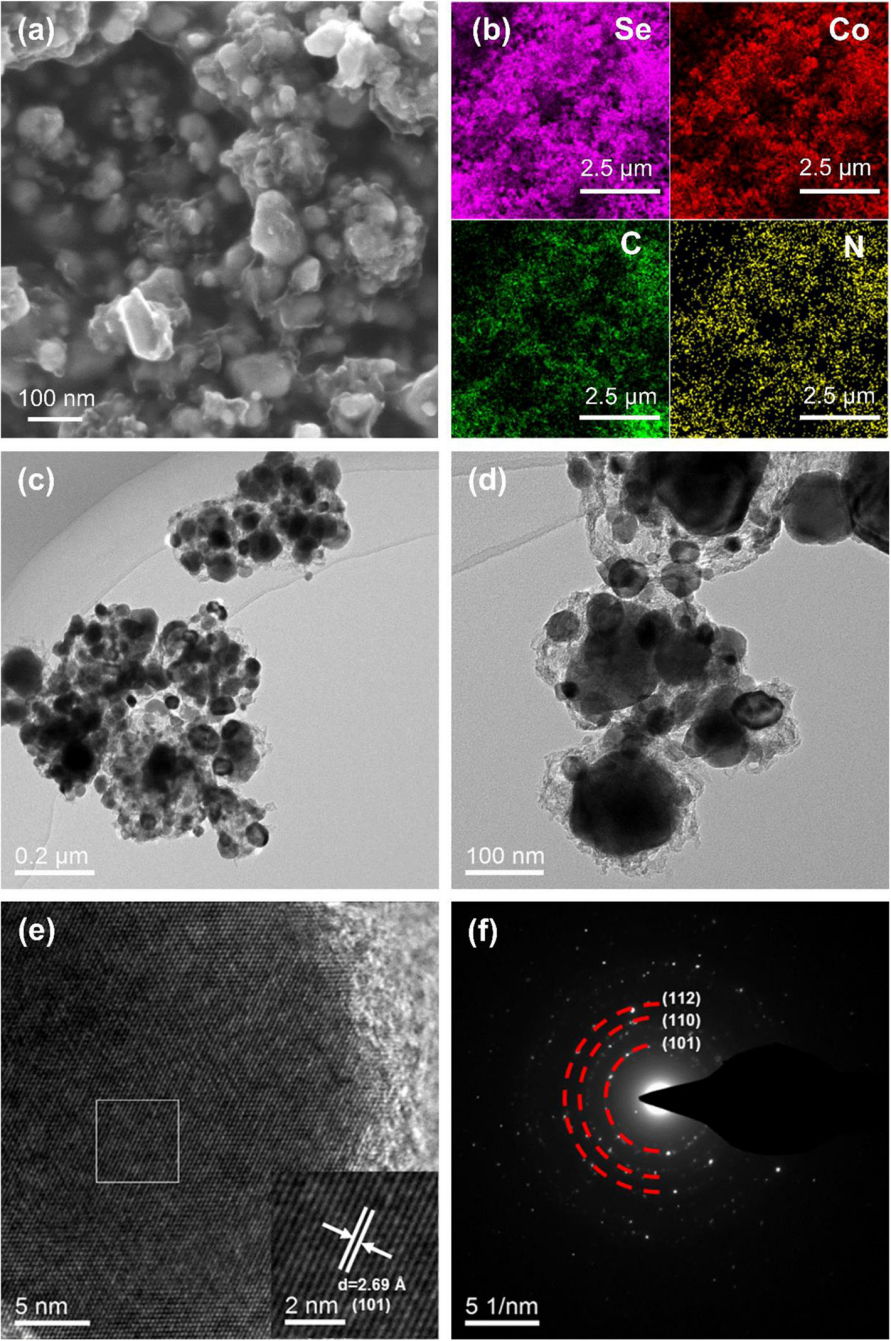
**a** SEM image of CoSe/NC. **b** Elemental mapping images of CoSe/NC. **c**, **d** TEM images of CoSe/NC. **e** HRTEM images of CoSe/NC. **f** SAED pattern of CoSe/NC

The morphology and microstructure of CoSe/NC were characterized through SEM and TEM. As shown in Fig. 5a, the SEM image of CoSe/NC presents that the CoSe nanoparticles are enfolded by NC with a size of 30–70 nm. However, the CoSe/NC cannot inherit the decahedral morphology from the precursor ZIF-67 because of the structure collapse and volume expansion, as previously mentioned. The energy dispersive spectrometer (EDS) results suggest the presence of Se, Co, C, N, and O in CoSe/NC, and the percentage of nitrogen is 0.27% (wt%) (Additional file 1: Figure S3 and Table S1). Besides, the elemental mapping images are shown in Fig. 5b, which suggest the uniform distribution of Se, Co, C, and N. The TEM image further discloses the structure, in which the coating structure of CoSe nanoparticles enwrapped with NC nanolayers could be clearly observed (Fig. 5c, d). In the coating structure, the NC nanolayers could preferably enhance the conductivity and restrain the volume expansion during the lithium insertion process. The HRTEM image exhibits a distinct interlayer spacing of 2.69 Å, which could be well indexed to the (101) lattice plane of CoSe (Fig. 5e). Meanwhile, note that the peripheral NC nanolayers are amorphous, which is consistent with the result of Raman. The SAED pattern shows several diffraction rings not spots, indicating the as-synthesized CoSe/NC is polycrystalline. These rings could match with the (101), (110), and (112) lattice planes of CoSe, which is supported by XRD (Fig. 5f).

**Fig. 6:**
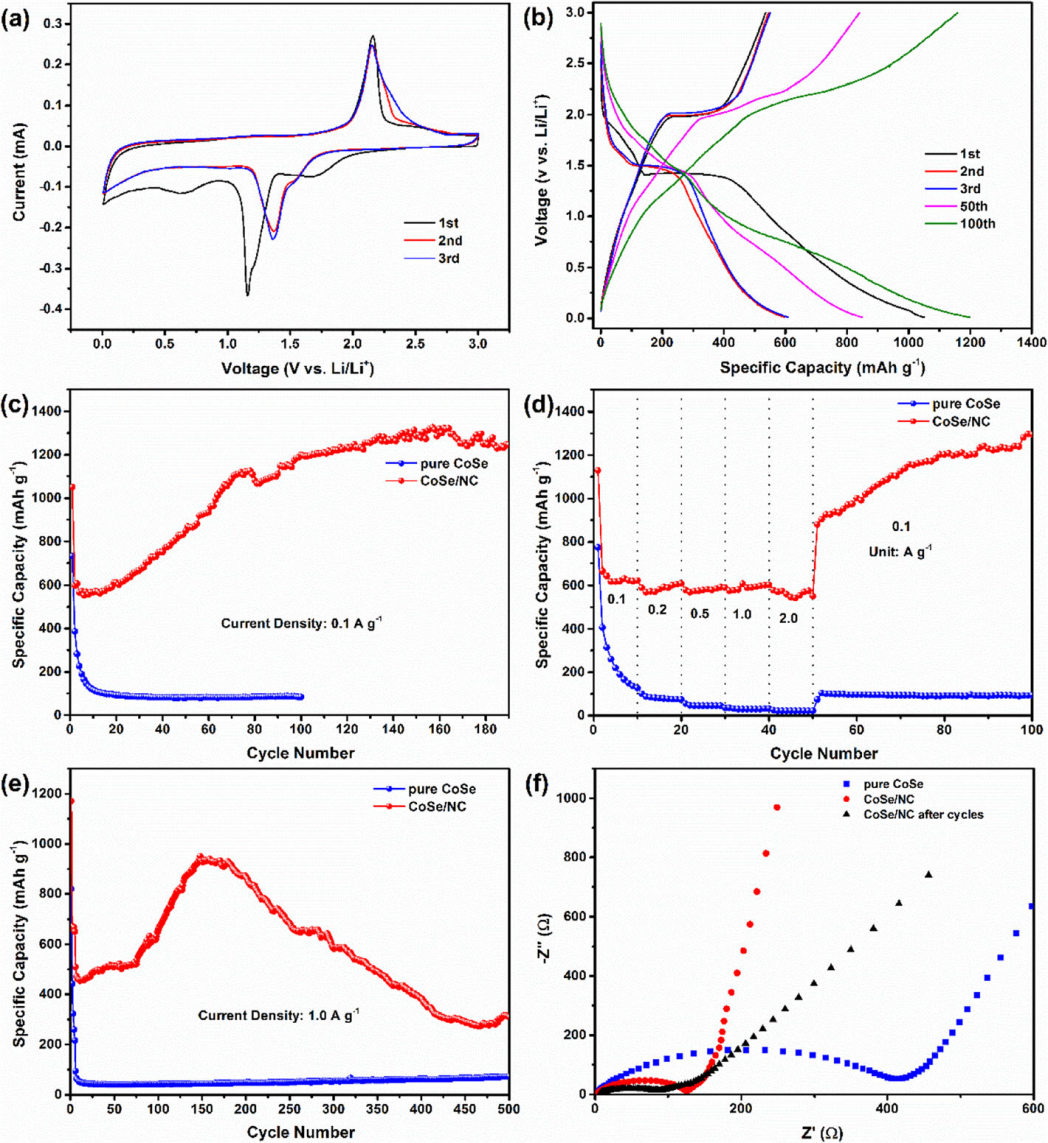
**a** The CV curves of CoSe/NC at 0.2 mV s^−1^. **b** Galvanostatic discharge/charge voltage profiles of CoSe/NC at 0.1 A g^−1^. **c** Cycling performance of CoSe/NC and pure CoSe at 0.1 A g^−1^. **d** Rate performance of CoSe/NC and pure CoSe at 0.1 to 1.0 A g^−1^. **e** Long-current cyclic performances of CoSe/NC and pure CoSe at 1.0 A g^−1^. **f** EIS spectras of pure CoSe, and CoSe/NC before and after cycling

To explore the electrochemical behavior of CoSe/NC, the CV curves were performed with a scan rate of 0.2 mV s^−1^ (Fig. 6a). As can be seen, there are a sharp reduction peak at 1.15 V and a weak hump at 0.64 V in the initial cathodic sweep, corresponding to the conversion of CoSe to Co and Li_2_Se and the constitution of SEI layer [22, 44, 45, 47]. As for the anodic sweep, the oxidation peak locates at 2.15 V that is ascribed to the formation of CoSe from Co and Li_2_Se, and overlaps well with those in the consecutive sweeps, suggesting the excellent cyclic stability. In the second and third cathodic sweeps, the reduction peaks shift to 1.37 from 1.15 V. The CV curves of pure CoSe show the similar characteristic with CoSe/NC (Additional file 1: Figure S4). To evaluate the lithium storage performance of CoSe/NC, the galvanostatic discharge/charge test was conducted. As shown in the galvanostatic discharge/charge curves of CoSe/NC at 0.1 A g^−1^, there are obvious discharge and charge platforms at 1.4 and 2.0 V, which are in agreement with the positions of reduction and oxidation peaks in abovementioned CV curves (Fig. 6b). Besides, the initial discharge and charge capacities are 1049.42 and 535.18 mAh g^−1^, separately, with a CE of 50.99%. The large irreversible capacity and low CE are ascribed to the constitution of SEI layer in the initial discharge process. It is noteworthy that the 100th discharge and charge capacities reach to 1199.34 and 1158.88 mAh g^−1^, which is larger than initial discharge capacity, and there are no evident platforms.

Figure 6c displays the cyclic property of CoSe/NC at 0.1 A g^−1^. It could be observed that there is a distinct incremental behavior of discharge capacity during the whole cycle, combined with the aforementioned no platforms in the 100th curves, which may be ascribed to pseudocapacitance that is a kind of redox reaction happening in the exterior of electrode materials [45, 47]. In the initial several cycles, the surface area for pseudocapacitance is less. In the subsequent cycles, the CoSe nanoparticles pulverized into small pieces caused by volume expansion. Therefore, there are more active sites for pseudocapacitance. Due to the CoSe nanoparticles are coated by NC nanolayers, the integrity of total structure is maintained. The CoSe/NC shows a first discharge capacity of 1049.42 mAh g^−1^. At 157th cycle, the specific capacity reaches the maximum value of 1325 mAh g^−1^. In the subsequent cycles, the specific capacity gradually decreases. After 190 cycles, it remains 1244 mAh g^−1^. The rate performance of CoSe/NC was evaluated at distinct current densities of 0.1, 0.2, 0.5, 1.0, and 2.0 A g^−1^ (Fig. 6d). After 10 cycles at above a series of current densities, the corresponding capacities are 623.21, 609.72, 590.34, 603.77, and 551.33 mAh g^−1^, separately. Notably, the current density rises from 0.1 to 2.0 A g^−1^, and the capacity merely declines 11.5%, indicating that CoSe/NC possesses exceptional rate performance. When the current density returns back to 0.1 A g^−1^, the capacity rapidly recovers to 880.09 mAh g^−1^ and gradually rises to 1295.81 mAh g^−1^ after 50 cycles, suggesting that the CoSe/NC still maintains the structural integrity even at 2.0 A g^−1^.

The lithium storage property of CoSe/NC at a large current density of 1.0 A g^−1^ was also performed, as shown in Fig. 6e. To generate homogeneous and compact SEI layer, CoSe/NC was cycle at 0.05 A g^−1^ for the initial 5 cycles. Generally, on the one hand, the large current density will lead to serious polarization behavior that limits the release of capacity; on the other hand, it will damage the structure of electrode materials that causes the speedy capacity fading. However, when the current density rises to 1.0 A g^−1^, the reversible capacity is 509.09 mAh g^−1^. In the subsequent cycles, the reversible capacity of CoSe/NC exhibits a growing behavior similar with those at 0.1 A g^−1^ and rate performance. In 148th cycle, the reversible capacity reaches to the maximum of 950.27 mAh g^−1^. After 500 cycles, CoSe/NC remains a relatively large reversible capacity of 310.11 mAh g^−1^. As for the pure CoSe, the reversible capacity is only about 72.75 mAh g^−1^, which is much lower than that of CoSe/NC. Such an outstanding lithium storage performance of CoSe/NC could be ascribed to NC nanolayers, which could provide additional capacity as active sites and enhance the conductivity, and the C–Se bonds between NC nanolayers and CoSe nanoparticles that could help NC nanolayers better buffer the strain caused by the volume change during cycling process.

Figure 6f shows the EIS of pure CoSe and CoSe/NC (before and after 100 cycles at 0.1 A g^−1^). The three Nyquist plots exhibit same features, a semicircle and an inclined line. The diameter of semicircle is related to the charge-transfer resistance and internal resistance. As can be seen, the semicircle in CoSe/NC is clearly smaller than that of CoSe, suggesting CoSe/NC possesses better electrical conductivity, which should be assigned to the NC nanolayers. Besides, after 100 cycles at 0.1 A g^−1^, the diameter of CoSe/NC declines than before, which may be associated with the constitution of SEI layer on the appearance of electrode materials and the pulverization of CoSe nanoparticles to increase the contact area between electrode and electrolyte.

**Fig. 7:**
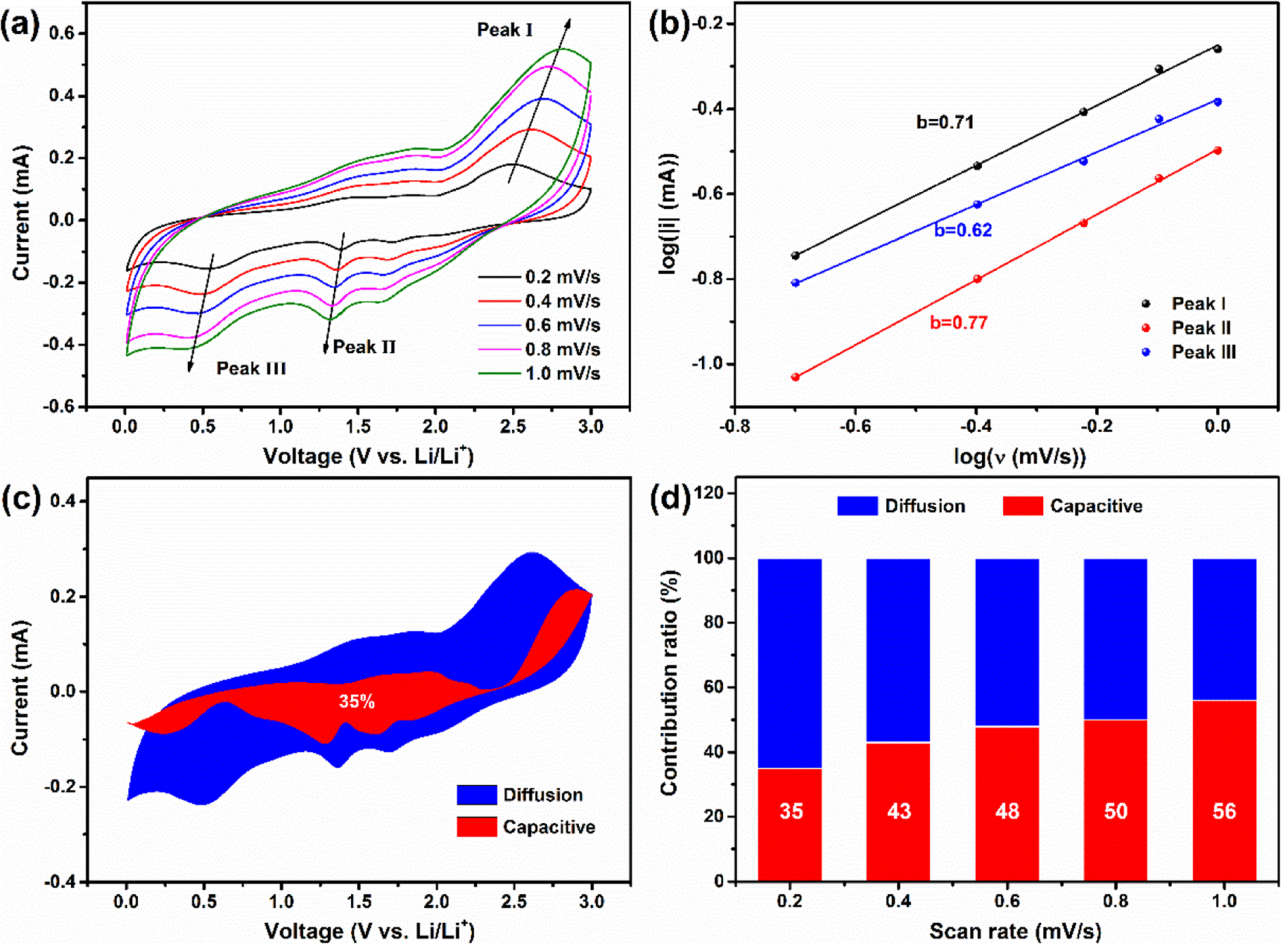
**a** CV curves of CoSe/NC after 100 cycles at 0.2 to 1.0 mV s^−1^. **b** Plots of log ν versus log |i| for the three peaks in CV curves. **c** Capacitive and diffusion-controlled contribution to charge storage at 0.2 mV/s. **d** Contribution ratio of capacitive and diffusion-controlled capacities at 0.2 to 1.0 mV s^−1^

To further investigate and analysis the capacity increase behavior of CoSe/NC, the electrochemical kinetics was conducted based on the CV measurements at various scan rates of 0.2 to 1.0 mV s^−1^ after 100 cycles at 0.1 A g^−1^ (Fig. 7a). According to the generation mechanism of capacity, the capacity could be divided into two types, diffusion-controlled capacity that is a kind of redox reaction in bulk phase (typical insertion/extraction of lithium ions), and capacitive-controlled capacity that includes double layer capacitance and pseudocapacitance. On the contrary, the capacitive-controlled capacity occurs on the surface of electrode materials, the double layer capacitance is a physical adsorption process, but the pseudocapacitance is a highly reversible redox reaction. In CV curves, the relevance between current (i) and scan rate (ν) could be represented through an equation, as follows [48–51]:

1 $$ i=a{\nu}^b $$

2 $$ \mathit{\log}i= blog\nu +\mathit{\log}a $$

where a and b are adjustable values. If *b* = 0.5, suggesting a diffusion-controlled process. However, the value of *b* is 1.0, corresponding to the capacitive-controlled process. To determine the value of *b*, Eq. (1) is transformed into Eq. (2). The value of *b* could be gained by computing the slope of log (ν) versus log (*i*). According to the above-mentioned method, the *b* values of peaks I, II, and III in CV curves are quantified to 0.71, 0.62, and 0.77, separately, suggesting that the capacity is contributed by a hybrid controlling process, as shown in Fig. 7b. The specific capacity contribution of two controlling process also could be obtained based on the following equation:

3 $$ i(V)={k}_1\nu +{k}_2{\nu}^{1/2} $$

4 $$ i(V)/{\nu}^{1/2}={k}_1{\nu}^{1/2}+{k}_2 $$

where *k*_1_ and *k*_2_ are adjustable values. The current (*i*) is composed of capacitive-controlled (*k*_1_*ν*) and diffusion-controlled process (*k*_2_*ν*^1/2^). In order to calculate the values of k_1_ν and *k*_2_*ν*^1/2^, Eq. (3) is converted into Eq. (4). The values of *k*_1_ and *k*_2_ correspond to the slope and intercept of i(*V*)/*ν*^1/2^ versus ν^1/2^, respectively. According to this method, *k*_1_*ν* and *k*_2_*ν*^1/2^ at various potential could be gained. As shown in Fig. 7c, the area represents the contribution ratio; the contribution of capacitive-controlled process is 37% at 0.2 mV s^−1^. The contribution ratios at other scan rates are also conducted (Fig. 7d). The contribution ratios of capacitive-controlled process are 43%, 48%, 50%, and 56% at 0.4, 0.6, 0.8, and 1.0 mV s^−1^, respectively. Above results demonstrate the electrochemical process of CoSe/NC is a hybrid controlling process.

## Conclusions

In summary, the original CoSe/NC composites were prepared by using ZIF-67 as precursor, in which the CoSe nanoparticles are coated by NC nanolayers and they are connected through C–Se bonds. In the as-prepared composites, the NC nanolayers could improve the conductivity of CoSe, buffer the volume expansion, and take part into the lithium storage reaction as active sits. The coating structure and C–Se bonds make the connection between CoSe nanoparticles and NC nanolayers closer, which are beneficial for the work of NC nanolayers to improve the electrochemical properties. Therefore, the CoSe/NC composites exhibit outstanding cyclic capability and rate performance as anode materials for LIBs. The CoSe/NC composites could deliver a reversible capacity of 1244 mAh g^−1^ after 190 cycles at 0.1 A g^−1^. Even at a large current density of 1.0 A g^−1^, it can remain 310.11 mAh g^−1^ after 500 cycles. Besides, according to the results of electrochemical kinetics, the electrochemical process of CoSe/NC is a hybrid controlling process. These results indicate that preparing metal compounds/carbonaceous composites by using MOFs, as precursor is valid strategy to enhance the lithium storage properties of metal compounds.

## Supplementary information

**Additional file 1. Figure S1**. SEM image of as-synthesized ZIF-67. **Figure S2**. C 1s spectrum of CoSe/NC. **Figure S3**. The EDS spectrum of CoSe/NC. **Figure S4**. CV curves of pure CoSe at 0.2 mV s^-1^. **Table S1**. Percentages of elements in CoSe/NC.

## Data Availability

All data are fully available without restriction.

## Abbreviations

BET: Brunauer-Emmett-Teller
BJH: Barrett-Joyner-Halenda
CV: Cyclic voltammetry
DMC: Diethyl carbonate
EC: Ethylene carbonate
EDS: Energy dispersive spectrometer
EIS: Electrochemical impedance spectroscopy
LIBs: Lithium-ion batteries
MOFs: Metal-organic frameworks
NC: N-doped carbon
PVDF: Polyvinylidene fluoride
TEM: Transmission electron microscopy
TGA: Thermogravimetric analysis
XPS: X-ray photoelectron spectroscopy
XRD: X-ray power diffraction

## References

[1] Liu Z, Yang S, Sun B, Chang X, Zheng J, Li X (2018). A peapod-like CoP@C nanostructure from phosphorization in a low-temperature molten salt for high-performance lithium-ion batteries. Angew Chem.

[2] Bai J, Xi B, Mao H, Lin Y, Ma X, Feng J, Xiong S (2018). One-step construction of N,P-codoped porous carbon sheets/CoP hybrids with enhanced lithium and potassium storage. Adv Mater.

[3] Fu Y, Wei Q, Zhang G, Sun S (2018). Advanced phosphorus-based materials for lithium/sodium-ion batteries: recent developments and future perspectives. Adv Energy Mater.

[4] Wu R, Wang DP, Rui X, Liu B, Zhou K, Law AWK, Yan Q, Wei J, Chen Z (2015). In-situ formation of hollow hybrids composed of cobalt sulfides embedded within porous carbon polyhedra/carbon nanotubes for high-performance lithium-ion batteries. Adv Mater.

[5] Jiang T, Bu F, Feng X, Shakir I, Hao G, Xu Y (2017). Porous Fe_2_O_3_ nanoframeworks encapsulated within three-dimensional graphene as high-performance flexible anode for lithium-ion battery. ACS Nano.

[6] Wu T, Zhang S, He Q, Hong X, Wang F, Wu X, Yang J, Wen Z (2017). Assembly of multifunctional Ni_2_P/NiS_0.66_ heterostructures and their superstructure for high lithium and sodium anodic performance. ACS Appl Mater Interfaces.

[7] Li Z, Xu Z, Tan X, Wang H, Holt CMB, Stephenson T, Olsen BC, Mitlin D (2013). Mesoporous nitrogen-rich carbons derived from protein for ultra-high capacity battery anodes and supercapacitors. Energy Environ Sci.

[8] Zheng F, Yang Y, Chen Q (2014). High lithium anodic performance of highly nitrogen-doped porous carbon prepared from a metal-organic framework. Nat Commun.

[9] Li X, Zheng S, Jin L, Li Y, Geng P, Xue H, Pang H, Xu Q (2018). Metal-organic framework-derived carbons for battery applications. Adv Energy Mater.

[10] Lu Y, Yu L, Lou XW (2018). Nanostructured conversion-type anode materials for advanced lithium-ion batteries. Chem.

[11] Guo W, Sun W, Wang Y (2015). Multilayer CuO@NiO hollow spheres: microwave-assisted metal–organic-framework derivation and highly reversible structure-matched stepwise lithium storage. ACS Nano.

[12] Huang J, Fang G, Liu K, Zhou J, Tang X, Cai K, Liang S (2017). Controllable synthesis of highly uniform cuboid-shape MOFs and their derivatives for lithium-ion battery and photocatalysis applications. Chem Eng J.

[13] Xia G, Liu D, Zheng F, Yang Y, Su J, Chen Q (2016). Preparation of porous MoO_2_@C nano-octahedrons from a polyoxometalate-based metal–organic framework for highly reversible lithium storage. J Mater Chem A.

[14] Yu X-Y, Yu L, Lou XW (2016). Metal sulfide hollow nanostructures for electrochemical energy storage. Adv Energy Mater.

[15] Xu X, Liu W, Kim Y, Cho J (2014). Nanostructured transition metal sulfides for lithium ion batteries: progress and challenges. Nano Today.

[16] Gao X, Wang B, Zhang Y, Liu H, Liu H, Wu H, Dou S (2019). Graphene-scroll-sheathed α-MnS coaxial nanocables embedded in N, S Co-doped graphene foam as 3D hierarchically ordered electrodes for enhanced lithium storage. Energy Storage Mater.

[17] Zhou Y, Tian J, Xu H, Yang J, Qian Y (2017). VS4 nanoparticles rooted by a-C coated MWCNTs as an advanced anode material in lithium ion batteries. Energy Storage Mater.

[18] Wang X, Kim H-M, Xiao Y, Sun Y-K (2016). Nanostructured metal phosphide-based materials for electrochemical energy storage. J Mater Chem A.

[19] Sun M, Liu H, Qu J, Li J (2016). Earth-rich transition metal phosphide for energy conversion and storage. Adv Energy Mater.

[20] Zhu P, Zhang Z, Hao S, Zhang B, Zhao P, Yu J, Cai J, Huang Y, Yang Z (2018). Multi-channel FeP@C octahedra anchored on reduced graphene oxide nanosheet with efficient performance for lithium-ion batteries. Carbon.

[21] Wang X, Sun P, Qin J, Wang J, Xiao Y, Cao M (2016). A three-dimensional porous MoP@C hybrid as a high-capacity, long-cycle life anode material for lithium-ion batteries. Nanoscale.

[22] Li Y, Xu Y, Wang Z, Bai Y, Zhang K, Dong R, Gao Y, Ni Q, Wu F, Liu Y, Wu C (2018). Stable carbon–selenium bonds for enhanced performance in tremella-like 2D chalcogenide battery anode. Adv Energy Mater.

[23] Liu D-H, Li W-H, Liang H-J, Lü H-Y, Guo J-Z, Wang J, Wu X-L (2018). Coaxial α-MnSe@N-doped carbon double nanotubes as superior anode materials in Li/Na-ion half/full batteries. J Mater Chem A.

[24] Jin J, Zheng Y, Kong LB, Srikanth N, Yan Q, Zhou K (2018). Tuning ZnSe/CoSe in MOF-derived N-doped porous carbon/CNTs for high-performance lithium storage. J Mater Chem A.

[25] Wei Z, Wang L, Zhuo M, Ni W, Wang H, Ma J (2018). Layered tin sulfide and selenide anode materials for Li- and Na-ion batteries. J Mater Chem A.

[26] Lu T, Dong S, Zhang C, Zhang L, Cui G (2017). Fabrication of transition metal selenides and their applications in energy storage. Coord Chem Rev.

[27] Guan BY, Yu XY, Wu HB, Lou XW (2017). Complex nanostructures from materials based on metal–organic frameworks for electrochemical energy storage and conversion. Adv Mater.

[28] Cao X, Tan C, Sindoro M, Zhang H (2017). Hybrid micro-/nano-structures derived from metal–organic frameworks: preparation and applications in energy storage and conversion. Chem Soc Rev.

[29] Tang H, Zheng M, Hu Q, Chi Y, Xu B, Zhang S, Xue H, Pang H (2018). Derivatives of coordination compounds for rechargeable batteries. J Mater Chem A.

[30] Li Y, Xu Y, Yang W, Shen W, Xue H, Pang H (2018). MOF-derived metal oxide composites for advanced electrochemical energy storage. Small.

[31] Li B, Wen H-M, Cui Y, Zhou W, Qian G, Chen B (2016). Emerging multifunctional metal–organic framework materials. Adv Mater.

[32] Cui Y, Li B, He H, Zhou W, Chen B, Qian G (2016). Metal–organic frameworks as platforms for functional materials. Acc Chem Res.

[33] Cheng N, Ren L, Xu X, Du Y, Dou SX (2018). Recent development of zeolitic imidazolate frameworks (ZIFs) derived porous carbon based materials as electrocatalysts. Adv Energy Mater.

[34] Wei G, Zhou Z, Zhao X, Zhang W, An C (2018). Ultrathin metal–organic framework nanosheet-derived ultrathin Co_3_O_4_ nanomeshes with robust oxygen-evolving performance and asymmetric supercapacitors. ACS Appl Mater Interfaces.

[35] Ma Y, He J, Kou Z, Elshahawy AM, Hu Y, Guan C, Li X, Wang J (2018). MOF-derived vertically aligned mesoporous Co_3_O_4_ nanowires for ultrahigh capacity lithium-ion batteries anodes. Adv Mater Interfaces.

[36] Yin D, Huang G, Zhang F, Qin Y, Na Z, Wu Y, Wang L (2016). Coated/sandwiched rGO/CoSx composites derived from metal–organic frameworks/GO as advanced anode materials for lithium-ion batteries. Chemistry.

[37] Yu L, Yang JF, Lou XW (2016). Formation of CoS2 nanobubble hollow prisms for highly reversible lithium storage. Angew Chem Int Ed.

[38] Ge X, Li Z, Yin L (2017). Metal-organic frameworks derived porous core/shellCoP@C polyhedrons anchored on 3D reduced graphene oxide networks as anode for sodium-ion battery. Nano Energy.

[39] Pan Y, Sun K, Liu S, Cao X, Wu K, Cheong W-C, Chen Z, Wang Y, Li Y, Liu Y, Wang D, Peng Q, Chen C, Li Y (2018). Core–shell ZIF-8@ZIF-67-derived CoP nanoparticle-embedded N-doped carbon nanotube hollow polyhedron for efficient overall water splitting. J Am Chem Soc.

[40] Jiao L, Zhou Y-X, Jiang H-L (2016). Metal–organic framework-based CoP/reduced graphene oxide: high-performance bifunctional electrocatalyst for overall water splitting. Chem Sci.

[41] Xia G, Su J, Li M, Jiang P, Yang Y, Chen Q (2017). A MOF-derived self-template strategy toward cobalt phosphide electrodes with ultralong cycle life and high capacity. J Mater Chem A.

[42] Meng J, Niu C, Xu L, Li J, Liu X, Wang X, Wu Y, Xu X, Chen W, Li Q, Zhu Z, Zhao D, Mai L (2017). General oriented formation of carbon nanotubes from metal–organic frameworks. J Am Chem Soc.

[43] Huang FK, Horton RC, Myles DC, Garrell RL (1998). Selenolates as alternatives to thiolates for self-assembled monolayers: a SERS study. Langmuir.

[44] Li J, Yan D, Lu T, Yao Y, Pan L (2017). An advanced CoSe embedded within porous carbon polyhedra hybrid for high performance lithium-ion and sodium-ion batteries. Chem Eng J.

[45] Zhou Y, Tian R, Duan H, Wang K, Guo Y, Li H, Liu H (2018). CoSe/Co nanoparticles wrapped by in situ grown N-doped graphitic carbon nanosheets as anode material for advanced lithium ion batteries. J Power Sources.

[46] Sheng Z-H, Shao L, Chen J-J, Bao W-J, Wang F-B, Xia X-H (2011). Catalyst-free synthesis of nitrogen-doped graphene via thermal annealing graphite oxide with melamine and its excellent electrocatalysis. ACS Nano.

[47] Hu H, Zhang J, Guan B, Lou XW (2016). Unusual formation of CoSe@carbon nanoboxes, which have an inhomogeneous shell, for efficient lithium storage. Angew Chem Int Ed.

[48] Tang Y, Zhang Y, Malyi OI, Bucher N, Xia H, Xi S, Zhu Z, Lv Z, Li W, Wei J, Srinivasan M, Borgna A, Antonietti M, Du Y, Chen X (2018). Identifying the origin and contribution of surface storage in TiO_2_(B) nanotube electrode by in situ dynamic valence state monitoring. Adv Mater.

[49] Wang J, Polleux J, Lim J, Dunn B (2007). Pseudocapacitive contributions to electrochemical energy storage in TiO_2_ (Anatase) nanoparticles. J Phys Chem C.

[50] Chao D, Zhu C, Yang P, Xia X, Liu J, Wang J, Fan X, Savilov SV, Lin J, Fan HJ, Shen ZX (2016). Array of nanosheets render ultrafast and high-capacity Na-ion storage by tunable pseudocapacitance. Nat Commun.

[51] Yuan T, Jiang Y, Sun W, Xiang B, Li Y, Yan M, Xu B, Dou S (2016). Ever-increasing pseudocapacitance in RGO–MnO–RGO sandwich nanostructures for ultrahigh-rate lithium storage. Adv Funct Mater.

