# Supplementary material for: ZIF-67-Derived CoSe/NC Composites as Anode Materials for Lithium-Ion Batteries
Source: Nanoscale Res Lett. 2019 Dec 2;14:358. doi: 10.1186/s11671-019-3194-5 (PMC6889125; doi:10.1186/s11671-019-3194-5)
Supplement: Supplementary file 1 — Additional file 1. Figure S1. SEM image of as-synthesized ZIF-67. Figure S2. C 1s spectrum of CoSe/NC. Figure S3. The EDS spectrum of CoSe/NC. Figure S4. CV curves of pure CoSe at 0.2 mV s-1. Table S1. Percentages of elements in CoSe/NC. [file 11671_2019_3194_MOESM1_ESM.docx]

**Supporting information**

**ZIF-67-derived CoSe/NC composites as anode materials for lithium-ion batteries**

Zongyang Li^1,2,3^, Lian ying Zhang^4^, Lei Zhang^5^*, Jiamu Huang^3^, Hongdong Liu^1,2^*

1. Chongqing Key Laboratory of Micro/Nano Materials Engineering and Technology, Chongqing University of arts and sciences, Chongqing 402160, PR China

2. Research Institute for New Materials Technology, Chongqing University of Arts and Sciences, Chongqing 402160, PR China
3. College of Materials Science and Engineering, Chongqing University, Chongqing 400045, PR China

4. Institute of Materials for Energy and Environment, Qingdao University, Shandong 266071, PR China
5. College of Life Science, Chongqing Normal University, Chongqing 401331, PR China

*Corresponding author: **lhd0415@126.com (Hongdong Liu)
 leizhang0215@126.com (Lei Zhang)**


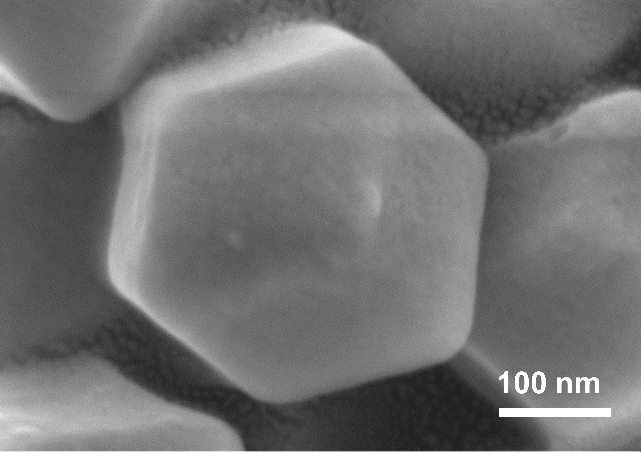


**Figure S1**. SEM image of as-synthesized ZIF-67.





**Figure S2**. C 1s spectrum of CoSe/NC.


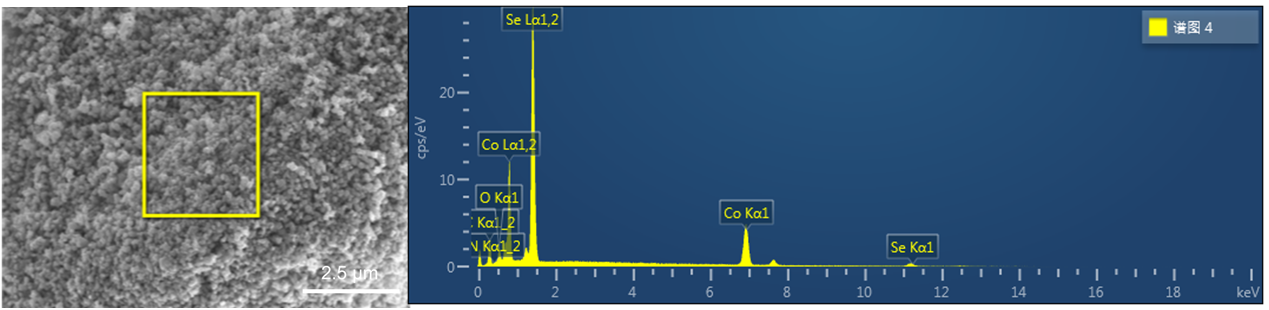


**Figure S3**. The EDS spectrum of CoSe/NC.

**Table S1.** Percentages of elements in CoSe/NC.

| elements | wt% |
| --- | --- |
| C | 18.46 |
| N | 0.27 |
| O | 2.77 |
| Co | 33.96 |
| Se | 44.54 |





**Figure S4**. CV curves of pure CoSe at 0.2 mV s^-1^.
